# The influence of selective mortality on poverty rates in later life: evidence from a Swedish cohort born in 1926

**DOI:** 10.1177/14034948241266437

**Published:** 2024-08-26

**Authors:** Johan Rehnberg, Olof Östergren, Ylva B Almquist, Johan Fritzell, Stefan Fors

**Affiliations:** 1 Aging Research Center, Karolinska Institutet and Stockholm University, Solna, Sweden; 2 Centre for Health Equity Studies (CHESS), Department of Public Health Sciences, Stockholm University, Sweden; 3 Center for Epidemiology and Community Medicine, Region Stockholm, Stockholm, Sweden

**Keywords:** Poverty, mortality, selective mortality, ageing

## Abstract

**Aims:**

Mortality influences the composition of the surviving population. Higher mortality among low-income individuals than high-income individuals may result in lower poverty rates in the surviving population. The objective of this study was to describe poverty rates for both survivors and deceased individuals in a cohort born in 1926.

**Methods:**

We used Swedish total population data on the 1926 birth cohort (*n* = 83,382), calculating annual poverty rates from 1991 to 2016. We compared poverty rates for the entire cohort, those who died the next year, and those who survived for 5, 10, or 20+ years, measuring the impact of selective mortality as the differences in poverty rates between the cohort and these subgroups.

**Results:**

Individuals who died the following year had higher poverty rates than the cohort at ages 65–90. Conversely, individuals who survived 5, 10, or 20+ years had lower poverty rates, with relatively small differences (1.1% to 6.9% lower) for survivors of 5 years or longer, and larger differences (26.4% to 32.8% lower) for those who survived 20+ years.

**Conclusions:**

Despite differences in mortality rates by income, selective mortality had only a modest impact on poverty rates. If life expectancy rises for all, and a more diverse population reaches old age, our findings indicate a potential slight increase in poverty rates due to reduced mortality as a selective factor. These findings emphasise the need to consider mortality selection when addressing future poverty rates in older adults.

## Introduction

There are substantial socioeconomic inequalities in longevity and mortality, with individuals at lower income levels more often experiencing premature death [1–3]. When individuals with lower incomes die at younger ages than those with higher incomes, a process of selection occurs, resulting in differences between the surviving population and the original population. In the case of income and poverty, the surviving population will exhibit higher income levels and lower poverty rates compared to the original population. The effect of mortality on the surviving population is often referred to in public health literature as selective mortality [4–6]. In this study, we aimed to quantify the extent to which selective mortality influences relative poverty rates and income levels among older persons.

The extent of relative income poverty among older European adults is substantial, as evidenced by the 2021 Pension Adequacy Report, which estimated that 16.1 million older persons in the European Union live below a relative poverty threshold [7]. When considering a global perspective, both absolute and relative income poverty are more common among older populations in less economically developed countries, often due to inadequate pension systems. Income patterns throughout the life course typically demonstrate a steady increase of income until middle age, reaching its peak between the ages of 40 and 60 [8,9]. Following retirement, income shifts from labour income to pension income, largely determined by prior labour market participation, and accumulated capital. The labour market exit coincides with a rise in poverty rates, individuals aged 80 and above experiencing the highest concentration of poverty across all age groups [10]. In Sweden, the prevalence of poverty among older adults exceeds that of the general population, particularly among older women [7,8,11,12].

As the population ages, welfare states face the challenge of maintaining pension systems that provide adequate income for older persons. If current relative poverty levels persist and more people enter old age, the number of older adults living in poverty will rise. In addition, with declining mortality rates, more low-income individuals may live longer, leading to a higher proportion of older people in poverty. Consequently, societal costs will increase due to both the growing number of older adults and the higher poverty rates among them.

One methodological approach used to investigate the influence of selective mortality is to compare trends between individuals who die with those who survive over a given period of time. This approach has been utilised in studies examining disability levels across age groups [13,14]. Findings from these studies demonstrate that the surviving population tends to have lower average disability levels compared with those who exited the population through mortality. This approach shows the compositional effects that mortality exerts on the surviving population. Building on this methodological framework, our study examined relative poverty rates in old age. We hypothesised that if selective mortality leads to the survival of a more socioeconomically advantaged population, we will observe lower poverty rates among the survivors. The disparity between the original population and the surviving population would in this case be driven by the disproportionate mortality experienced by individuals living with lower incomes.

This study aimed to describe relative poverty rates for both survivors and deceased individuals in Sweden after the age of 65 by analysing a cohort of individuals born in 1926 and following them over a period of 25 years. First, we considered the short-term impact of selective mortality by comparing the poverty in the original cohort to a subsample of individuals who died the following year. Second, we investigated the long-term impact of selective mortality on poverty rates by comparing the poverty rate in the original cohort with subsamples of the cohort that survived for 5, 10, and 20 years.

## Data and methods

The data used in this study were compiled from Swedish population registers. Yearly income information from 1991 to 2016 was retrieved from the Swedish Income and Tax Register [15]. Mortality information was obtained from the Cause of Death Register. Data were selected for one cohort of individuals born in 1926, who were 65 years old in 1991. Individuals who immigrated or emigrated before 1991 were excluded. In total, 83,382 individuals were observed. The use of data for the purposes of this study was granted by the Swedish Central Ethical Review Board (Dnr Ö 25-2017).

### Income and poverty

Income was measured yearly using equivalised disposable household income, an aggregate variable encompassing all after-tax income sources such as work, transfer payments, and income from capital. Zero or negative incomes were excluded from the analysis. To account for variations in household size and composition, we equivalised income by dividing the disposable household income by a factor determined by the household composition. In order to ensure comparability between the income data and the poverty thresholds retrieved from Statistics Sweden, we used the same equivalence factor in this study as the one employed by Statistics Sweden [16] (see Supplementary Table 1). By applying these adjustments, an income per capita measure was derived, accounting for the economic benefits of sharing living expenses within households.

Poverty rates were computed for each year from 1991 to 2016. To determine whether a person lived above or below the poverty level we compared household income levels to a yearly poverty threshold set at 60% of the median income. This definition of relative poverty is commonly used, for example in official statistics from the European Union [17]. We collected the median income level from publicly available income statistics provided by Statistics Sweden; the income measure used was equivalised disposable household income including capital gains [18]. The equivalence scale used by Statistics Sweden was used in this study for calculating disposable household income [16].

### Methods

To quantify the influence of mortality on poverty rates, we calculated poverty rates for the entire cohort and for subsamples of the cohort based on their date of death. In the first step we calculated the poverty rates for individuals who died the following year, resulting in poverty rates for each age between 65 and 90 for individuals who died at that age plus 1 year. For example, at age 70 we calculated the poverty rate for individuals who died the following year, when they were 71. By comparing the cohort poverty rate to the subsample that died the following year, we were able to assess the magnitude of the effect that selective mortality exerted on the cohort during a year.

In the second step, we investigated the long-term influences of selective mortality by taking subsamples of the cohort at each age that survived for 5 years or more, 10 years or more, and 20 years or more after a certain age. Through this process we obtained poverty rates for each age between 65 and 90 for individuals who survived that age plus an additional 5, 10, or 20 or more years. By comparing the poverty rates between subsamples of survivors and the entire cohort at each age, we assessed the impact that long-term selective mortality had on poverty rates as the cohort aged.

## Results

**Figure 1.**
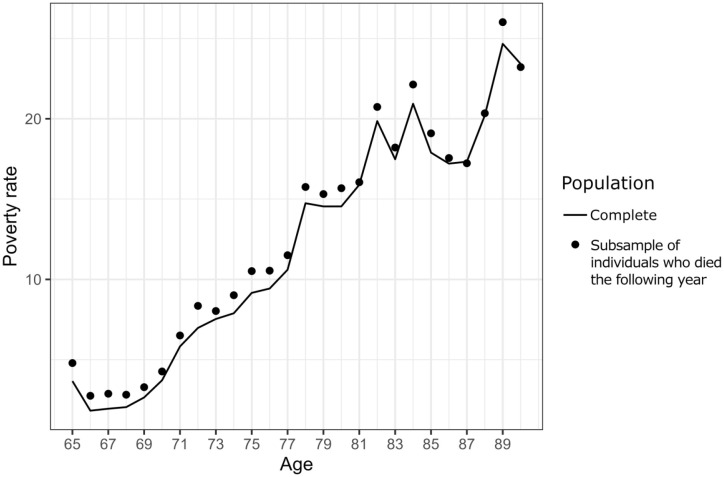
Poverty rate in the entire cohort (line) and among individuals who died the following year (points) from year 1991 to 2016 (aged 65–90).

Figure 1 (solid line) shows the poverty rate in the cohort at any given age, spanning from the initial measurement year in 1991, when individuals were aged 65, to the final year in 2016, when the cohort reached the age of 90. The dots represent the poverty rate for a subsample of individuals who died the subsequent year. For instance, the data point for individuals aged 65 illustrates the poverty rate for a subset of individuals who died at the age of 66 the following year.

In Figure 1, the dots are above the line at all ages, indicating that individuals who died the following year had a higher poverty rate than that observed in the entire cohort at the same age. Table I (column: Died the next year) presents the relative difference in poverty rates between the cohort and the subset of individuals who died the subsequent year. The largest relative difference between the poverty rate of the cohort and the poverty rate of individuals who died within the following year was observed among individuals younger than 70, ranging from 20% to 39% higher poverty rates among individuals who died. However, above the age of 70, the difference in poverty rates between the cohort and individuals who died the following year gradually diminished.

**Table I.** Relative difference (%) in poverty rates between the cohort poverty rate and individuals who died the following year (age +1), and between the cohort poverty rate and individuals who survived 5, 10, and 20 years or longer beyond the specific age category.

Age	Died the next year, %	Survivors for . . . years, %		
		5	10	20
65	27.4	–4.4	–10.6	–26.4
66	39.4	–5.0	–12.5	–32.8
67	27.5	–5.3	–10.5	–27.7
68	46.5	–6.9	–13.6	–32.8
69	19.7	–5.0	–9.6	–27.8
70	12.2	–4.9	–9.3	–29.9
71	23.6	–4.2	–8.8	
72	20.6	–3.0	–6.0	
73	13.6	–2.4	–5.9	
74	11.2	–2.0	–6.0	
75	7.8	–1.1	–2.6	
76	1.3	–2.2	–4.2	
77	2.0	–1.7	–3.3	
78	0.1	–1.4	–4.8	
79	1.6	–1.2	–4.4	
80	3.5	–1.8	–4.7	
81	5.8	–1.3		
82	5.4	–2.3		
83	0.3	–2.4		
84	1.7	–1.5		
85	6.2	–1.6		
86	3.8			
87	6.3			
88	1.4			
89	1.9			
90	–0.9			

**Figure 2.**
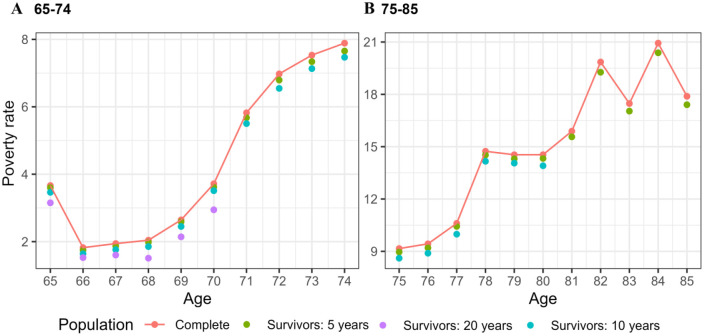
Poverty rate in the entire cohort (solid line) and among individuals who survived 5, 10, and 20 years beyond the specific age category. Note that the y-axis is not fixed.

Figure 2 (solid line) again shows the poverty rate in the cohort, this time divided into two age groups: first from the age of 65 to 74 (Figure 2a), followed by the 75 to 85 year group (Figure 2b). The dots in Figure 2 show the poverty rate for individuals who survived for 5-, 10-, and 20 years or more after reaching a specific age. For example, for individuals aged 70 in 1996, the cohort poverty rate was 3.7%. However, for individuals who survived 5 years or more after turning 70, the poverty rate was 4.9% lower compared with the cohort’s rate at age 70 (see Table I column: Survivors for 5 years). The poverty rates from which Figure 2 is plotted are presented in Supplementary Table 2.

Individuals who survived 10 years or longer after the age of 70 had a 9.3% lower poverty rate, and those who survived for 20 years or more had a 29.9% lower poverty rate compared with the cohort poverty rate at the age of 70. The overall trend indicated that individuals who survived for longer periods tended to experience lower poverty rates than the poverty rate observed in the entire cohort at the same age. The impact of mortality selection was greater below the age of 70, gradually diminishing as individuals aged (see Table I). The magnitude of the selective effects indicated that mortality selection occurring after 5 years had a small to moderate impact on cohort poverty rates, ranging between 1.1% and 6.9%. The impact of selective mortality was more substantial when examining the longer selection periods of 10 years and 20 years.

## Discussion

The future economic circumstances of the older population remain uncertain. Factors such as disparities in labour market conditions and potential inadequacies in future pension schemes may contribute to increased old-age poverty rates in the coming years [19]. In addition to demographic and institutional influences on income standards and poverty rates, processes of mortality selection may affect the composition of the surviving population that reaches old age. Research on life expectancy and mortality consistently demonstrates that individuals with lower incomes tend to have shorter lifespans [1,20]. Based on this understanding, we hypothesised that for any given age, individuals who died soon after the income measurement would exhibit higher poverty rates than the poverty rates observed in the complete cohort. Similarly, we expected a subsample of individuals who survived for longer periods of time to have lower poverty rates than the rates observed in the full cohort. To examine this, we observed the poverty rate in one cohort born in 1926 that were followed from the age of 65 in year 1991 to age 90 in the year 2016.

The findings revealed that individuals who died 1 year after being observed experienced higher poverty rates compared with same aged cohort members. In addition, individuals who survived for longer periods experienced lower poverty rates. These results provide support for the notion that selective mortality suppresses poverty rates among older adults. However, the influence that mortality had on the composition of the surviving population was small to moderate in size. Among individuals who survived for 5 or 10 years beyond a certain age, the poverty rate was at most 5% to 10% lower compared with the cohort poverty rate at the same age.

The extent to which mortality can impact poverty rates and income levels by altering the population composition relies on two factors: the correlation between mortality and income, and the number of deaths that occur. For instance, in the working-age population, where there is a strong social gradient in mortality [21–23] but a low mortality rate, the limited number of deaths will exert a minimal influence on the composition of the surviving population. In contrast, most deaths occur in the older population above the age of 70. However, the income gradient in mortality decreases substantially after the age of 75 and becomes negligible among individuals aged 85 or older [24]. As a result, the age bracket spanning from 65 to 75 presents the highest potential for mortality to reshape the surviving population. In these ages the income gradient in mortality is substantial and mortality rates are relatively high. In support of these previous observations, our results showed that the most pronounced impact of selective mortality on the composition of the surviving population occurred in ages below 75.

The lower impact of mortality on poverty rates in older ages was most likely a result of the weakened association between income and mortality that has been previously observed. It has been proposed that other factors start to play a more prominent role in determining mortality outcomes among the oldest old [25–27]. These factors may include age-related health conditions, access to healthcare, and individual resilience. Therefore, while mortality still exerts some influence on poverty rates among the oldest survivors, its impact is attenuated compared with younger age groups due to the complex interplay of various factors in determining mortality outcomes in advanced ages.

Projections indicate an anticipated increase in life expectancy at birth by nearly 2 years in 2030 in Sweden, accompanied by an increase in the number of individuals aged 75 and above, estimated to be around 700,000 [28]. This, in combination with our finding that those who survived consistently experienced lower poverty rates, suggests that there is a potential for higher old-age poverty rates in future cohorts of older adults. This scenario aligns with the failure-of-success hypothesis, which suggests that medical advancements have allowed more vulnerable individuals to reach old age, possibly leading to increased rates of morbidity and mortality within certain age groups [6]. This perspective is rarely, if ever, considered in analyses of old-age poverty.

One limitation of the current study is that to observe the long-term impact that selective mortality has on poverty rates, we needed to have mortality data many years after the time period when poverty rates were observed, which limited the possibility of including more recent cohorts of older adults. Despite the narrow focus on one birth cohort in the data, we believe that the overall results are generalisable to any population where an income gradient in mortality is present. However, the extent of the influence that mortality has on the surviving population will be dependent on the context-specific age–mortality patterns and the strength of the income gradient in mortality.

A common problem when examining income and mortality lies in the possibility that income decreases prior to death due to health-related issues preceding mortality. This phenomenon is often referred to as selective mortality, or conversely, the health worker effect, where individuals actively engaged in the labour force tend to be in better health. However, such problems are unlikely to have any substantial impact on the cohort that we studied in this paper. Given that they reached the age of 65 in 1991, prior to the introduction of a flexible retirement system in Sweden, very few individuals continued working beyond the age of 65 at that time.

## Conclusions

This study aimed to describe relative poverty rates for both survivors and deceased older individuals in Sweden. The findings revealed that selective mortality affected poverty rates, albeit of a small to moderate magnitude. Considering a future scenario characterised by increases in life expectancy for the entire population, and where a more diverse range of individuals reaches old age, our results suggest the potential for slightly elevated poverty rates in the older population. Projections or policy plans that fail to account for this are likely to underestimate the future rates of old-age poverty.

## Supplemental Material

sj-docx-1-sjp-10.1177_14034948241266437 – Supplemental material for The influence of selective mortality on poverty rates in later life: evidence from a Swedish cohort born in 1926 Supplemental material, sj-docx-1-sjp-10.1177_14034948241266437 for The influence of selective mortality on poverty rates in later life: evidence from a Swedish cohort born in 1926 by Johan Rehnberg, Olof "math170-014233122412stergren, Ylva B Almquist, Johan Fritzell and Stefan Fors in Scandinavian Journal of Public Health
