# Supplementary material for: The influence of selective mortality on poverty rates in later life: evidence from a Swedish cohort born in 1926
Source: Scand J Public Health. 2024 Aug 26;53(7):782–7. doi: 10.1177/14034948241266437 (PMC12598060; doi:10.1177/14034948241266437)
Supplement: sj-docx-1-sjp-10.1177_14034948241266437 – Supplemental material for The influence of selective mortality on poverty rates in later life: evidence from a Swedish cohort born in 1926 [file sj-docx-1-sjp-10.1177_14034948241266437.docx]

**Supplementary material:** Supplementary data

**Title:** The influence of selective mortality on poverty rates in later life: evidence from a Swedish cohort born in 1926

**Content**

Supplementary table 1-2

**Supplementary table 1.** Household composition adjustment score

| Number of persons in the household | Factor |  |
| --- | --- | --- |
| One person households and the first adult in larger households | 1.00 |  |
| Second adult | 0.51 |  |
| First child (age 0-19) | 0.52 |  |
| Second and each additional children (age 0-19) | 0.42 |  |
| Each additional adult | 0.60 |  |

**Supplementary table 2.** Poverty rate in the cohort, poverty rate for individuals who died the following year (age + 1), and poverty rate for individuals who survived 5, 10, and 20 years or longer beyond the specific age category.

|  |  |  | Survivors for, years | | |
| --- | --- | --- | --- | --- | --- |
| Age |  | Died next year | 5 | 10 | 20 |
| 65 | 3.7% | 5.0% | 3.5% | 3.3% | 2.9% |
| 66 | 1.8% | 3.0% | 1.7% | 1.6% | 1.4% |
| 67 | 1.9% | 2.7% | 1.8% | 1.8% | 1.5% |
| 68 | 2.0% | 3.8% | 1.9% | 1.8% | 1.5% |
| 69 | 2.6% | 3.3% | 2.5% | 2.4% | 2.1% |
| 70 | 3.7% | 4.2% | 3.5% | 3.4% | 2.9% |
| 71 | 5.8% | 7.6% | 5.6% | 5.4% |  |
| 72 | 7.0% | 8.8% | 6.8% | 6.6% |  |
| 73 | 7.5% | 8.7% | 7.4% | 7.1% |  |
| 74 | 7.9% | 8.9% | 7.7% | 7.4% |  |
| 75 | 9.2% | 9.9% | 9.1% | 8.9% |  |
| 76 | 9.4% | 9.6% | 9.2% | 9.1% |  |
| 77 | 10.6% | 10.8% | 10.4% | 10.3% |  |
| 78 | 14.7% | 14.8% | 14.5% | 14.1% |  |
| 79 | 14.5% | 14.8% | 14.4% | 13.9% |  |
| 80 | 14.5% | 15.1% | 14.3% | 13.9% |  |
| 81 | 15.9% | 16.9% | 15.7% |  |  |
| 82 | 19.9% | 21.0% | 19.4% |  |  |
| 83 | 17.5% | 17.5% | 17.1% |  |  |
| 84 | 20.9% | 21.3% | 20.6% |  |  |
| 85 | 17.9% | 19.1% | 17.6% |  |  |
| 86 | 17.2% | 17.9% |  |  |  |
| 87 | 17.3% | 18.5% |  |  |  |
| 88 | 20.2% | 20.5% |  |  |  |
| 89 | 24.7% | 25.1% |  |  |  |
| 90 | 23.4% | 23.2% |  |  |  |
